# Acute effects of exercise on macro- and microvasculature in individuals with type 1 diabetes – a secondary outcome analysis

**DOI:** 10.3389/fendo.2024.1406930

**Published:** 2024-08-29

**Authors:** Adam Saloň, Karin Schmid-Zalaudek, Bianca Steuber, Alexander Müller, Othmar Moser, Suhaila Alnuaimi, Per Morten Fredriksen, Benedicta Ngwenchi Nkeh-Chungag, Nandu Goswami

**Affiliations:** ^1^ Division of Physiology & Pathophysiology, Otto Loewi Research Centre for Vascular Biology, Immunology, and Inflammation, Medical University of Graz, Graz, Austria; ^2^ Faculty of Applied Ecology, Agricultural Sciences and Biotechnology, Inland Norway University of Applied Sciences, Hamar, Norway; ^3^ Vascular Biology Center, Augusta University, Augusta, GA, United States; ^4^ Trials Unit for Interdisciplinary Metabolic Medicine, Division of Endocrinology and Diabetology, Department of Internal Medicine, Medical University of Graz, Graz, Austria; ^5^ Exercise Physiology & Metabolism, Institute of Sports Science, University of Bayreuth, Bayreuth, Germany; ^6^ College of Medicine, Mohammed Bin Rashid University of Medicine and Health Sciences, Dubai, United Arab Emirates; ^7^ Faculty of Health, Welfare and Organization, Østfold University College, Fredrikstad, Norway; ^8^ Department of Biological and Environmental Sciences, Faculty of Natural Sciences, Walter Sisulu University, Mthatha, South Africa; ^9^ Integrative Health Department, Alma Mater Europeae, Maribor, Slovenia

**Keywords:** diabetes mellitus, type 1, exercise, microcirculation, haemodynamics, vascular stiffness

## Abstract

**Background:**

Type 1 diabetes is a chronic autoimmune disease associated with insulin-producing beta cell destruction, declining insulin secretion, and elevated blood glucose. Physical activity improves glycaemic control and cardiovascular health. This study explores acute effects of maximal exhaustion induced by a cardiopulmonary exercise on macro- and microvascular parameters in type 1 diabetes.

**Methodology:**

Twenty-five participants with type 1 diabetes (14 males, 11 females), aged 41.4 ± 11.87 years, BMI 23.7 ± 3.08, completed a repeated-measure study. Measurements pre-, post-, 30- and 60-minutes post-exhaustion involved a maximal incremental cardio-pulmonary exercise test. Macro- and microvascular parameters were assessed using VICORDER^®^ and retinal blood vessel image analysis. Repeated measures ANOVA in SPSS (Version 27.0) analysed data.

**Results:**

Post-exercise, heart rate increased (p<.001), and diastolic blood pressure decreased (p=.023). Diabetes duration correlated with pulse wave velocity (r=0.418, p=.047), diastolic blood pressure (r=0.470, p=.023), and central retinal arteriolar equivalent (r=0.492, p=.023).

**Conclusion:**

In type 1 diabetes, cardiopulmonary exercise-induced exhaustion elevates heart rate and reduces diastolic blood pressure. Future research should explore extended, rigorous physical activity protocols for greater cardiovascular risk reduction.

## Introduction

Type 1 diabetes (T1D) is a chronic autoimmune disease in which the immune system attacks the insulin-producing beta cells of the pancreas, thereby significantly reducing the body’s insulin secretion, leading to increasing blood glucose levels and risk for cardiovascular disease (CVD) (1, 2). CVDs are the main cause of morbidity and mortality in patients suffering from T1D (3, 4). In individuals with diabetes, there are alterations in the microvasculature (which plays an important role in maintaining blood pressure (BP) and the efficient delivery of nutrients throughout the body)., particularly affecting the capillary basement membrane in various organs such as the glomeruli, retina, myocardium, skin, and muscles. These changes can appear even prior to the onset of hyperglycaemia and vascular pathologies and gradually contribute to the development of complications associated with diabetes (5–7). The progressive alterations in the microvasculature not only impact vessel functionality but also give rise to clinical issues like hypertension. Indeed, abnormalities in retinal microvasculature as well as microvasculature are two important markers of subclinical CVDs, which have been demonstrated to be impaired in individuals with T1D (8–10). Physical activity exerts hemodynamic forces on the arterial wall, including an increase in intravascular pressure, which in turn stimulates shear stress. Shear stress plays an essential role in the production of nitric oxide (NO), a basis of the beneficial effects of physical activity on vascular health. The enhanced production of NO contributes to reduced systemic vascular resistance, peripheral vasodilation, and improved vascular blood flow. These effects have a strong impact on vascular health promoting optimal cardiovascular function. Thus, physical activity serves among other things as a powerful tool for preventing and treating cardiovascular risks and diseases, including those associated with diabetes.

However, physical inactivity is prevalent among adults with T1D due to the potential side effects of hypoglycaemia, which prevents these individuals from engaging in physical activity. These side effects include dizziness, confusion, shakiness, sweating, weakness, and in severe cases, loss of consciousness or seizures. Such symptoms can impair coordination and concentration, reduce physical performance and endurance, and increase the risk of accidents or injuries during exercise. Consequently, the fear of experiencing hypoglycaemia-related symptoms can lead to anxiety and avoidance of physical activity among individuals with T1D. Nonetheless, prior research indicates favourable cardiovascular outcomes associated with extended periods of exercise in people diagnosed with T1D (11). The acute effects of physical activity on arterial stiffness and retinal microvasculature in individuals with T1D are, however, not well understood. Previous studies have reported conflicting results regarding the effect of physical activity on pulse wave velocity (PWV) in individuals with T1D (12–15). While some studies did not detect any changes in PWV (12), others observed an improvement in PWV as a result of exercise (13–15). Despite these findings, this area of research remains underinvestigated. To validate the results observed in these smaller studies, more large cohort studies are needed to provide conclusive evidence regarding the impact of physical activity on PWV in T1D individuals. Similarly, the effect of physical activity on retinal microvasculature has been studied in individuals with diabetes in general but not specifically in those with T1D (6, 7, 16–19). While cross-sectional cohort studies link diabetes and arteriolar widening; the prospective long duration studies connect diabetes with arteriolar narrowing (6, 7, 16–19). The aim of this secondary outcome study was to investigate the acute effects of maximum exhaustion induced by a cardiopulmonary exercise (CPX) on PWV and retinal microvasculature in individuals with T1D. PWV and retinal microvasculature were measured pre-, following-, and 30 and 60 minutes after- the CPX.

## Methodology

This repeated measure study was performed at the Interdisciplinary Metabolic Medicine Trials Unit, Clinical Division of Endocrinology and Diabetology of the Medical University of Graz, Austria. This randomized single-centre, cross over ULTRAFLEXI-1 trial, was approved by the local ethics committee (31-551 ex 18/19) and registered at the German Clinical Trials Register (DRKS00018065; drks.de). All data have been collected according to good clinical practices and compliant with the Declaration of Helsinki of WMA (2013). All participants received detailed information about the study protocol and signed written consent about participation in it.

### Sample size calculations

Since this manuscript describes the secondary outcomes of a crossover ULTRAFLEXI study, the sample size calculation reflects that of the primary study (20). It was determined using a paired t-test (5%, two-sided alpha), showing that a sample size of 20 would have 80% power to detect a difference in means of 100 ± 150 minutes between IGlar-U300 and IDeg-U100. To achieve balance in the eight sequence groups, the aim was to include n=24 patients, giving a power of around 87%. Considering the occurrence of potential study dropouts, a maximum of 25 participants were planned to be included in this trial.

### Study participants

In this study, 25 individuals with T1D, aged 18-65 years were enrolled. The participants had been diagnosed with type 1 diabetes mellitus for at least 1 year with an HbA1c ≤ 10% and a c-peptide level <0.3nmol/L. Participants were required to be treated with multiple insulin injections, had a body mass index (BMI) of 18.0 – 29.9 kg/m^2^ and a VO_2peak_ of >20 ml·kg-1·min^-1^. Participants receiving systemic corticosteroid, nonselective beta-blocker, or growth hormone treatment were excluded. Additionally, individuals with, heart failure classified as NYHA III or IV, angina pectoris, recent myocardial infarction (within the last 12 months), advanced retinopathy, neuropathy, hypoglycaemia unawareness, or an estimated glomerular filtration rate (eGFR) less than 50 mL/min/1.73 m² (20) were also excluded.

### Study protocol

**Figure 1:**
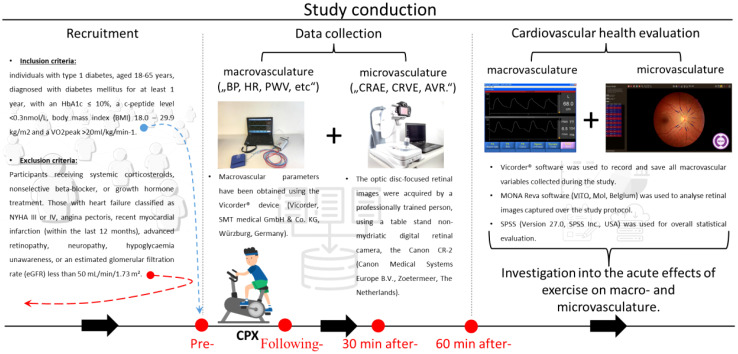
A brief overview of the study. A horizontal line, at the bottom of the image, accompanied by black arrows pointing rightward illustrates the study’s temporal progression. The “Recruitment” section provides foundational details about inclusion/exclusion to/from the study. The “Data collection” section is displayed through photos of devices to assess (micro-) macrovascular parameters. In addition, the bottom part shows the measurement protocol, with a CPX pictogram and red dots, highlighting the time points of the data collection. The final, “Cardiovascular health evaluation” section is introduced via the VICORDER® and MONA Reva software used for collecting and analyzing vascular variables. It also emphasizes the study objective of investigation into the acute effects of exercise on macro- and microvasculature. Cardiopulmonary exercise (CPX), Blood Pressure (BP), Heart Rate (HR), Pulse Wave Velocity (PWV), Central Retinal Arteriolar Equivalent (CRAE), Central Retinal Venular Equivalent (CRVE), and Artery to Vein Ratio (AVR).

This secondary analysis constitutes a component of the ULTRAFLEXI-1 study, which had the primary objective of evaluating glycaemic control two basal insulins, insulin degludec and insulin glargine U300 at two different dosages in people with T1D performing moderate exercise sessions with regard to glycaemic control (20). In a subset of participants, parameters of vascular function were measured at four-time points; pre-, following-, and 30 and 60 minutes after- the CPX testing, which took part prior to the regular exercise sessions. The simplified overview of the study protocol can be seen in **Figure 1**. In addition to vascular measurements, this secondary analysis of the ULTRAFLEXI-1 study presented also additional data, such as TDD (total daily dose) and Hba1c (haemoglobin A1C), collected within the main project ((20); **Table 1**).

**Table 1:** Characteristics of study participants population.

Parameter	Baseline characteristics of the study participants
N (males/females)	25 (14/11)
Age [years]	41.4 (11.87)
Height [m]	1.76 (0.10)
Weight [kg]	74.41 (15.34)
BMI [kg/m^2^]	23.72 (3.08)
TDD [U]	41.52 (16.75)
Hba1c [mmol/mol]	59.72 (8.97)
HbA1c [%]	7.62 (0.82)
Diabetes duration [years]	16.76 (10.41)
Hemodynamics and arterial stiffness at baseline	
Heart rate [bpm]	67.76 (12.54)
Systolic blood pressure [mmHg]	134.04 (14.37)
Diastolic blood pressure [mmHg]	71.13 (6.79)
Pulse wave velocity [m/s]	7.67 (1.70)
Microvascular parameters at baseline	
CRAE [mm]	148.16 (18.33)
CRVE [mm]	213.39 (22.27)
AVR	0.70 (0.06)

The data are presented as mean (standard deviation) unless otherwise stated. BMI (body mass index), TDD (total daily dose), HbA1c (haemoglobin A1C), CRAE (central retinal artery equivalent), CRVE (central retinal vein equivalent), AVR (artery to vein ratio).

### Exercise protocol

The Cardiopulmonary Exercise (CPX) test was selected for this study due to its well-established safety and standardization, especially for individuals with chronic conditions like Type 1 Diabetes (T1D). The CPX test offers a balanced approach that rigorously assesses participants’ cardiopulmonary fitness while prioritizing their comfort and safety. This test is instrumental in precisely measuring key parameters such as VO2 max, ventilatory thresholds, and oxygen uptake kinetics, which are crucial for a comprehensive understanding of the cardiopulmonary fitness of the participants. Its use ensures reproducibility and comparability with existing literature, thereby providing a reliable baseline for future research.

The CPX test was conducted using a 1-minute step protocol, with step increments tailored to the participant’s gender and fitness level at 10, 15, or 20 watts, continuing until maximum exhaustion was reached. Maximum exhaustion was defined as the point where the cadence dropped below 60 revolutions per minute, with the average duration of the step tests being 12.9 ± 2.4 minutes. Following the achievement of maximum load, characterized by high acidosis, participants underwent an active cool-down phase consisting of low-intensity movement at 20 watts. This cool-down phase lasted for 6 minutes, and measurements were taken immediately afterward to ensure the safety and well-being of the participants.

### Macrovascular measurements

All macrovascular parameters were collected by the VICORDER^®^ (Vicorder, SMT medical GmbH & Co. KG, Würzburg, Germany), similar to our previous studies (21, 22).

The study participants were in a supine position in a quiet room, with relaxed skin and muscles over the carotid. The measurements began with BP readings as their values are necessary for measuring PWV. The BP values were measured using the VICORDER^®^ when the inflating cuff was placed on the left upper arm. One measurement per person, per session (pre-, following-, and 30 and 60 minutes after- the CPX) was performed corresponding to the ESH/ESC guidelines.

PWV was obtained by placing the small inflating cuff over the right carotid and another, bigger one, over the right thigh. Before starting the measurement, the length between the carotid cuff and thigh cuff was determined (23). Then the measurements were started and lasted until carotid and thigh pressure waveforms were clear and reproducible. The length between the cuffs of the carotid and thigh measured during the baseline measurements was used during the latter measurements.

### Microvascular measurements

A tabletop, non-mydriatic digital retinal camera Canon CR-2 (Canon Medical Systems Europe B.V., Zoetermeer, The Netherlands) was used to capture retinal fundus images (22, 24). Optic disc-focused retinal images (resolution of 1536 × 1536) of the right eye were obtained from each of the participants during each time point of the study. The images were always collected after measuring macrovascular parameters.

Subsequently, the data were stored, organized, and prepared for evaluation of microvascular parameters. A single grader, blinded from study details used the semi-automated MONA REVA software (VITO, Mol, Belgium (25);) to analyse the retinal fundus images. The grader has a year of experience in retinal image analysis and is certified in retinal image analysis and has participated in numerous research projects involving retinal imaging in both clinical and research settings. This extensive experience and certification ensure the accuracy and reliability of the image evaluations. The scale ratio, also known as the resolution number, was calculated by averaging the individual resolution numbers from all retinal images.

Retinal images were processed automatically and vessel diameter analysis in zone 0.5 to 1 of the optic disc diameter from the margin of the optic disc, were performed by the Mona Reva software. The post-processing steps, such as double thresholding, blob extraction, the removal of small, connected regions, and filling holes, followed. Following these steps, the grader checked the quality and correctness of analysed vessel assignments, potentially corrected them, and labelled vessels as arterioles or venules.

The six largest retinal arterioles and the six largest retinal venules corresponding to the Parr–Hubbard–Knudtson formula were used for calculations (26). The central retinal artery equivalent (CRAE), and central retinal vein equivalent (CRVE) were obtained and expressed in micrometres (μm). Additionally, the ratio between CRAE and CRVE was computed to obtain the artery-to-vein ratio (AVR).

### Statistical analysis

All the vascular changes were analysed using repeated measures of ANOVA, with timepoint (1-4) as the repeated factor. Data distribution was examined, and cases that substantially deviated from the group mean (± 3 standard deviations) were excluded from further analysis (n=1 for TORS). Biographic data such as age, BMI or duration of diabetes were correlated with (micro-) macrovascular variables by Pearson correlation. Furthermore, the participants were median split by the duration of diabetes (“diabetes long”: 25.3 ± 6.60 years, n=13; “diabetes short”: 7.5 ± 3.06 years, n=12) to compare the effect of duration on diabetes on vascular parameters (**Table 1**). Statistical analysis was performed by SPSS (Version 27.0, SPSS Inc., USA).

## Results

The present study included twenty-five individuals with T1D (14 males, 11 females) with a mean age of 41.4 ± 11.87 years (range: 20-62), and a mean BMI of 23.7 ± 3.08. The basic characteristics of the study participants are summarized in **Table 1**. Complete data sets encompassing all from both (micro-) macrovascular parameters across all four measurement points of the study were available from only 13 participants. This reduction was primarily due to participant attrition, as some participants were unable to attend all four measurement sessions due to personal scheduling conflicts, medical reasons, or other commitments. Additionally, technical difficulties such as poor image quality or equipment malfunction at certain measurement points contributed to incomplete data collection.

**Figure 2:**
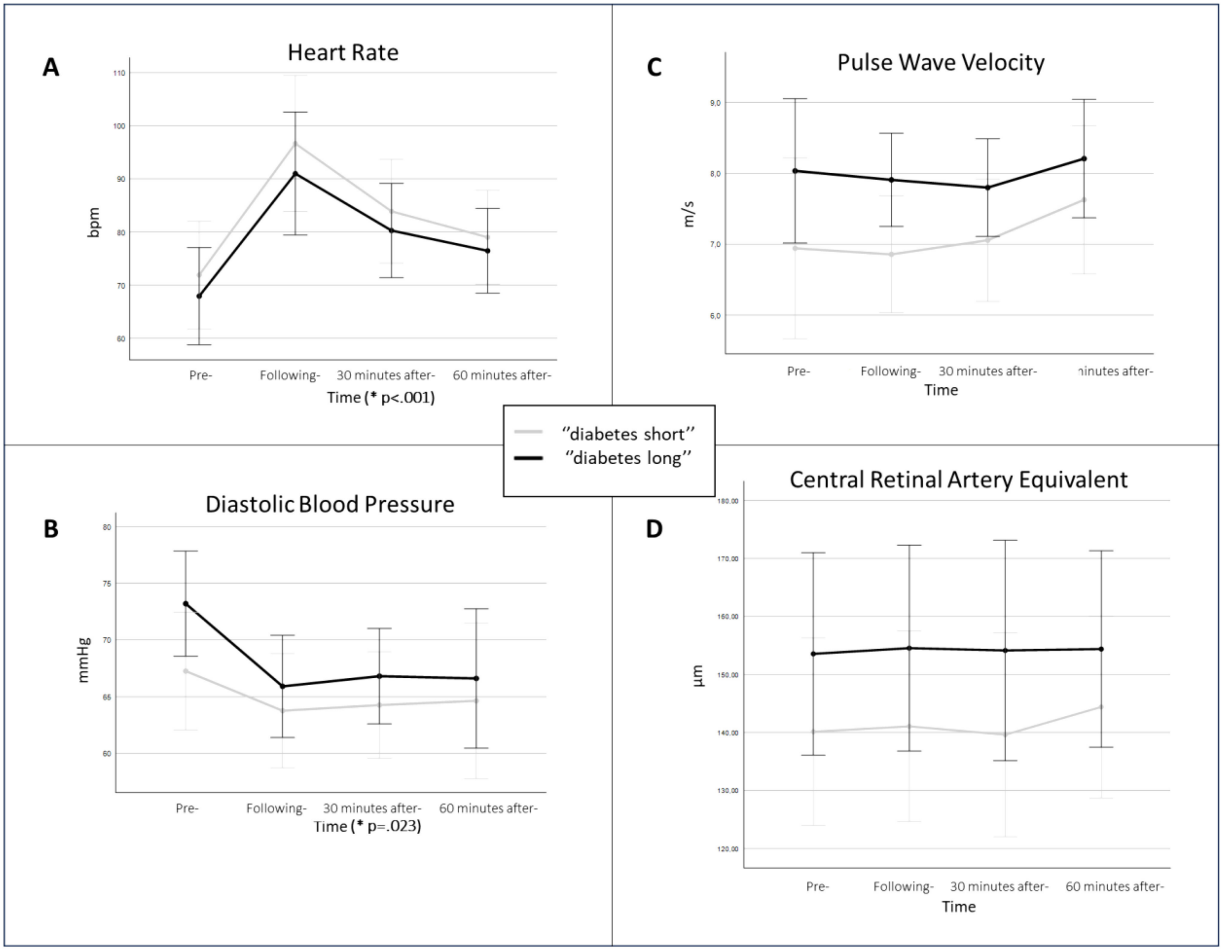
The picture displays (micro-) macrovascular parameters in the study population split into two groups by the duration of diabetes. The study individuals were median-split by the duration of diabetes (“diabetes long”, black line; “diabetes short”, grey line). It compares **(A)** heart rate, **(B)** diastolic blood pressure, **(C)** pulse wave velocity, and **(D)** central retinal artery equivalent; over the four measurement points of the study protocol. The p-value is presented only in **(A)** heart rate and **(B)** diastolic blood pressure, as for the other parameters no significant difference was observed. The p-value is displayed on the time axis, to highlight that the time was the effect of significance. The data is presented by means of the given measurement time points with respective confidence intervals.

An elevation in heart rate (HR) and reduction in diastolic BP was observed (**Figure 2**). Additionally, at baseline, diastolic BP and PWV from macrovascular and CRAE from microvascular parameters were correlated with the duration of diabetes.

### Hemodynamics and arterial stiffness at baseline (macrovascular assessment)

A significant elevation in HR was observed over the four measurement points (F (1.7;30.1) =25.759, p<.001) (**Figure 2**). Although the individuals with shorter diabetes duration exhibited higher HR values, there was no significant difference between diabetes duration groups (F (1,18) =.464, p=.505).

The analysis of baseline data showed a correlation between diastolic BP and the duration of diabetes (r=0.470, p=.023). Additionally, the significant reduction in diastolic BP was observed over the four measurement points (F (1.8,29) =4.499, p=.023) (**Figure 2**). Furthermore, even though the individuals with longer diabetes duration consistently, over the study protocol, exhibited a greater decrease and higher diastolic BP values, there was no captured significant difference in diabetes duration groups (F (1,16) =1.229, p=.284) (**Figure 2**).

Similarly, as with diastolic BP, the analysis of baseline data showed a correlation between PWV and the duration of diabetes (r=0.418, p=.047), and there was a tendency towards higher PWV values during the study protocol in patients with longer duration of diabetes (**Figure 2**). However, no significant changes in PWV were captured, neither between the four measurement points (F (1.7,27) =1.715, p=.202) nor diabetes duration groups (F (1,16) =2.764, p=.116).

### Microvascular measurements

CRAE at baseline was correlated to the duration of diabetes (r=0.492, p=.023). When comparing microvascular parameters across all four measured time points, there were no significant differences, neither in CRAE over time (F (2.7,29) =.993, p=.403) nor between the two diabetes duration groups (F (1,11) =1.411, p=.260). However, participants with longer duration of diabetes exerted consistently higher mean CRAE values (**Figure 2**). Additionally, there were no significant changes noted in CRVE, neither over time (F (1.9,21.1) =1.678, p=.211) nor between the two groups with different diabetes duration (F (1,11) =.868, p=.371). Similarly, to CRVE, no significant differences were observed in AVR across the entire protocol.

## Discussion

The present study investigated the acute effect of high-intensity cardiopulmonary exercise in individuals with diabetes type 1. While observing significant elevation in HR and reduction in diastolic BP after the CPX, no effect was observed with other vascular function parameters. The splitting of study participants into two groups based on the duration of diabetes showed that duration positively correlated with PWV, diastolic BP, and CRAE (**Figure 2**).

Only a handful of studies have investigated the effects of physical activity in T1D patients on macrovascular variables as was carried out in the present study. However, the reported results from these studies are inconsistent thus continuing the discussion regarding a crosslink between physical activity and T1D patients. As the T1D population is frequently young, when it commonly appears in childhood and adolescence, a lot of studies have been conducted on the young population. The inclusion of the participants in the present study, with a mean age of 41.4 (SD: 11.87) overcame generalizations and perceive problems from a broader perspective compared to conclusions from only young population studies. Furthermore, physical activity programs are usually investigating long-term effects, rather than focusing on short-term ones as in the present study. Therefore, short-term setup of the present study, compared to previous studies, supports to balance of the research topic. Despite these above-mentioned differences between previous research and the present study, the following discussed studies are closely matched with the setup of the present study. It is important to remember that while physical activity generally improves the cardiovascular profile (12–17, 27–34), the influence of diabetes exerts the opposite (6, 7, 12–15, 17–19, 27–29).

### Heart rate

The study of Ansell and colleagues investigated high-intensity physical activity in T1D vs non-T1D individuals (38 minutes, treadmill, elicited ~90% of maximal HR in four 4-minute segments) (13). They showed that HR during the final minute of these segments reached 82%, 90%, 92%, and 90% of peak HR, while the subsequent recovery segments elicited 64%, 68%, 69%, and 65% of peak HR, respectively (13). HR in the control group remained significantly above baseline values also in the postexercise period. The T1D group showed 17% higher HR values at 10 minutes postexercise compared to baseline, however, it reached baseline values with longer postexercise time (13). The present study contains only the T1D group and the CPX test lasted much shorter, when the participants in our study faced maximal exhaustion, while the study above elicited ~90% of maximal HR in four 4-minute segments. Despite, the differences between them and the present study, the present study similarly observed a significant elevation in HR as the effect of the CPX test (from Pre- to Following-) which followed a gradual decline (from Following- to 30 minutes after- and 60 minutes after-). To support these findings, the study conducted by Gusso and colleagues, which implemented a 20-week (four 60-minute exercise sessions per week) physical activity, including the individuals with and without T1D, observed a significant reduction in resting HR in both training groups (29). Contrary to the above, Brazeau and colleagues explored a 12-week training program in T1D patients (aged 44.6 ± 13.3 years) and did not capture significant changes in HR (28).

### Blood pressure

A cross-over study of Way and colleagues on the adult diabetic population confirmed the effect of physical activity when they compared 3 groups [1.) high-intensity interval physical activity (cycling for 4 × 4 min at 85%–95% of HR peak, 2.) moderate-intensity continuous physical activity (33 min of continuous cycling at 60%–70% of HR peak, and 3.) control (lying quietly in a supine position for 30 min)] (12). They found a significant group × time effect in transient systolic BP reduction and diastolic BP elevation for the high-intensity interval physical activity group (12). In another study, Ansell and colleagues observed an increase in both BPs when comparing baseline measurements to 105 min post-physical activity, however, they did not see any significant changes in BP between T1D and non-T1D individuals (13). Compared to the above studies, the present study observed an unexpected reduction in diastolic BP as an acute effect of the CPX test. Similarly, to us, they investigated the short-term effect of exercise. However, the study group in our study faced maximal exhaustion because of CPX, while their study protocol contained 4 repeated high-intensity bouts, which could be the main reason for observed discrepancies. The study of Scott and colleagues comparing the effect of two types of training [6 weeks; 3 sessions per week; 1.) high-intensity cycling (HIT): every session contained multiple bouts of HIT, 2.) moderate-intensity cycling (MICT): 30-50 minutes of MICT] in T1D individuals did not note any significant changes in BP (15). Brazeau and colleagues captured favourable reductions in both, systolic and diastolic BP in T1D patients after 12-week training program (28). Similarly, Gusso and colleagues observed a reduction in submaximal exercise systolic BP and resting diastolic BP in T1D individuals after 20 weeks of training (29). The longest study we discuss is the one conducted by Salem and colleagues. This study compared three different groups (no-exercise, exercise sessions once/week, exercise sessions three times/week) before and after 6 months (35). They also concluded the exercise-related beneficial effects by reducing diastolic BP in the third mentioned study group (35). Like these studies, our study observed a reduction in diastolic BP. However, they investigated the effect of long-term implementation of a physical activity program that could lead to physiological-morphological adjustments in the cardiovascular system, while the observed, unexpected acute reduction in diastolic BP seen in the present study after short, one-time maximal exhaustion caused by CPX, could be easily caused only by naturally occurring fluctuations in BP. Furthermore, the present study also noted a positive correlation between diabetes duration and diastolic BP at baseline, proposing disease progression-dependent vascular damage.

### Pulse wave velocity

A previously mentioned studies conducted either by Way and colleagues or by Ansell and colleagues did not, similarly to the present study, observe changes in PWV (12, 13). Despite the similarity that they investigated the short-term effects of exercise, our study differs from theirs. Our study group experienced maximal exhaustion due to CPX, while their study protocol included 4 repeated high-intensity bouts. The short duration exercise in theirs or CPX in our study may be the reason that prevented us from capturing significant changes. It is possible, that prolonged physical activity programs must be implemented to transfer the effect into changes in arterial compliance in relatively healthy individuals with T1D. Two other studies investigated the effect of longer period of physical activity and observed that physical activity was associated with a lower PWV (14, 15, 27). The length of the protocols and a higher load of physical activity in their studies may be the reason for observed changes in PWV, which were not captured in the present study. Moreover, rather than a short-term, acute effect of physical activity observed in the present study, their study protocol captured physical activity-related chronic vascular adaptations. Furthermore, the short time of physical activity in the present study may not reveal the changes in arterial compliance in relatively healthy individuals with T1D. Finally, the present study showed a positive correlation between PWV at baseline and the duration of diabetes that could highlight possible T1D duration-dependent damage in the cardiovascular system.

### Microvascular parameters

As it was recently reviewed by Hanssen and colleagues, the associations of retinal vessel diameters with diabetes appear heterogeneous (17). Diabetes is associated with both narrowing (7) and widening (18, 19) of retinal arteries (CRAE). Furthermore, an association between diabetes and the widening of retinal venules (CRVE) was demonstrated (6).

While cross-sectional cohort studies link diabetes and arteriolar widening; the prospective long duration studies connect diabetes with arteriolar narrowing (6, 7, 17–19). The present study observed that CRAE at baseline was correlated to the duration of diabetes. Persistent inflammation and rising oxidative stress during hyperglycaemia are associated with pericyte loss (36, 37). Hyperglycaemia impairs myogenic constriction of retinal arterioles (38, 39) leading to arteriolar widening as a short-term consequences of disease observed in the cross-sectional cohort studies (6, 18, 19). On the other hand, the narrowing of retinal arteries seems to represent a more advanced stage of retinal microvascular impairment and indicates a longer-term consequence of the disease (7). Based on this information, the correlation between CRAE at baseline and diabetes duration observed in the present study showed opposite results. The explanation for why we did not see narrowing in retinal arteries as a long-term consequence of diabetes could lie in the low sample size as well as in the type of diabetes (present study: T1D, most of the previous studies: T2D). Inflammation in diabetes, combined with factors like disease duration and patient age, can contribute to wider retinal venules. A meta-analysis conducted by Liu and colleagues supports the correlation between inflammation markers and retinal venular widening (40). The presented study did not detect any changes in CRVE, when the arguments mentioned above can serve as reasons, we also did not measure any inflammatory markers, which prevented us from this evaluation.

As there are no studies available to examine the interrelation between T1D, physical activity, and retinal microvasculature, this paragraph above discussed only the interconnection between retinal microvasculature and diabetes. The following paragraph will talk about the interconnection between physical activity and retinal microvasculature that will allow us to cross these three topics together at the end of the discussion.

A systematic review from Streese and colleagues investigated the interrelation between physical activity and retinal vessel diameter (41). They concluded, that higher physical activity is associated with microvascular profile improvement, specifically narrower CRVE and wider CRAE in both children as well as adults (41). While physical activity improves the retinal microcirculation profile (wider CRAE, narrower CRVE), physical inactivity plays the opposite (30–33).

Hanssen and colleagues observed the effect of regular physical activity (10 weeks) in 3 groups (n=15, obese athletes; n=14, lean amateur athletes, and n=17 lean elite athletes) (30). The study showed an increase in AVR in all groups, an increase in CRAE in obese athletes, and a reduction in CRVE in amateur athletes (30). Siegrist and colleagues showed that an 18-month school-based prevention program (JuvenTUM 3) caused an increase in AVR and CRAE, and reduction in CRVE, predominantly found in overweight and obese children (31). Another school-based physical activity program (20-minute physical activity sessions performed on 5 days a week over 8 weeks) further validated the physical activity-based improvement in the retinal microvasculature by higher CRAE (32). Streese and colleagues showed that high-intensity interval training (12-week Nordic Walking, three times per week) improved retinal microvasculature (wider CRAE, narrower CRVE, increased AVR) in individuals (mean age 59 ± 7 years) with increased cardiovascular risk (33). The present study did not observe any direct effect of CPX on retinal parameters. The duration of their study protocols was much longer compared with the present study, and maybe changes in retinal microvasculature need more time to emerge in relatively healthy individuals with T1D. Furthermore, the present study included much fewer participants and evaluated the effect of one-time maximal exhaustion caused by CPX, which is very different from the above studies. Moreover, our study included the T1D patients, compared to the healthy participants in their studies. Finally, the present study showed a positive correlation between CRAE at baseline and the duration of diabetes, suggesting disease duration-related remodelling of retinal vasculature.

In addition, the two prominent studies, the Multi-Ethnic Study of Atherosclerosis (MESA) and the Atherosclerosis Risk in Communities (ARIC) study which have explored various aspects, including the association between physical activity levels and retinal microcirculation parameters, demonstrated similar findings in this regard (16, 34). Both studies found that either higher levels of physical activity or lower levels of sedentary behaviour were associated with an improved retinal microvascular profile, consistent with the results observed in our study.

## Summary

To summarize the results of the present study, a significant elevation in HR as the effect of the CPX test (from Pre- to Following-) which followed a gradual decline (from Following- to 30 minutes after- and 60 minutes after-) was observed. The reduction in diastolic BP as an acute effect of CPX was unexpected and contrary to previous research. As a result of increased cardiovascular demand and oxygen absorption from working muscles, an increase in BP is expected during or shortly after exercise. We assume that these discrepancies could be anchored in a short and not-so-vigorous exercise protocol as can be seen in previous research. Therefore, diastolic BP reduction in the present study may only reflect naturally occurring BP-related fluctuations rather than the effect of CPX. Moreover, even though the sample size of the present study is small, the observed positive correlation between diabetes duration and diastolic BP as well as PWV at baseline, may suggest T1D duration-dependent damages in the cardiovascular system. Furthermore, this correlation remained consistent across all four measurements of the study. However, these suggestive results should be confirmed by future research.

As mentioned above, there are no studies that focused on the interconnection between T1D, physical activities, and microvascular changes. Previous research has suggested that while diabetes may potentially lead to vascular damage, a broad spectrum of different physical activities as well as their durations report improvements in it including improvements in retinal microcirculation. According to the previous research, the duration of physical activity matters, and the short duration as well as not-so-vigorous physical activity in the present study are considered the main drivers of the failure to capture significant changes in not only microvasculature parameters. Moreover, as we saw in diastolic BP and PWV, when dividing T1D individuals into two groups based on the diabetes duration, a positive correlation between diabetes duration and CRAE was observed. Similarly, as in macrovasculature, this correlation remained consistent across all four measurements of the study. Although many might believe that narrowing rather than widening of retinal arteries represents endothelial damage, the cohort studies (6, 18, 19) previously show, that hyperglycaemia, inflammation, and oxidative stress impair myogenic constriction of retinal arterioles (38, 39) leading to arteriolar widening as a short-term consequence of diabetes. However, because the correlation of the present study showed the opposite when the individuals suffering from T1D longer had wider retinal arterioles, these results should be confirmed or denied in future research.

## Limitations

A small sample size is one limitation of our study. However, our study holds importance in investigating the feasibility of such a study. Because of the low sample size, we decided not to sex-stratify the participants as it would significantly reduce the statistical power and could lead to irrelevant results. In addition, the participants have not been stratified based on disease severity, although T1D individuals were categorized according to diabetes duration to partially address this limitation. While splitting the participants based on disease duration to investigate its specific impact on cardiovascular parameters is valuable and important, the lack of statistical correction for age differences between the short and long-disease duration groups must be acknowledged. Although the duration of the disease was the main focus of our analysis, we recognize that age and exercise capacity are important confounding variables that can influence cardiovascular parameters.

Another limitation of our study is the absence of a healthy control group for direct comparison with the T1D cohort. This decision was influenced by logistical constraints, including time, funding, and participant recruitment efforts, which were beyond the scope of our current resources. Instead, we relied on established normative data from the literature to contextualize our findings. Future research will aim to include healthy control groups to provide a more comprehensive comparative analysis and to further validate our results.

Even though we standardized the endpoint of CPX using a maximal exhaustion test to limit variability, this approach does not eliminate the potential effects of age and fitness level on our results. Future studies should consider including parameters directly indicative of fitness ability, such as VO2 max, and performing statistical corrections for age to better account for these variables.

Although we were able to measure immediately after the CPX test, but it was a disadvantage that we could not measure directly after the maximum exhaustion of the CPX test. Nevertheless, the present study keeps its importance in need to investigate the feasibility of such a study. The study employed reliable and well-accepted methods for assessing (micro-) macrovasculature. Despite the robust methodology, it is worth noting that the evaluation of retinal images was performed by a single grader, introducing the potential for inter-rater variability. However, we believe this limitation is mitigated as the grader received specific training from the staff of MONA Reva.

## Conclusions and future directions

The present study showed that a maximal exhaustion induced by a CPX test in individuals with T1D results in significant elevation in HR and reduction in diastolic BP. However, this unexpected reduction in diastolic BP may only reflect naturally occurring BP-related fluctuations rather than the effect of CPX. Moreover, no other vascular variables were captured to change because of CPX. Therefore, these results must be confirmed or corrected in future research. However, longer durations of diabetes suggested more damaged vasculature, as indicated by positive correlation between diabetes duration and PWV, diastolic BP or CRAE.

Future studies employing more strenuous and longer physical activity protocols are recommended to better capture changes in cardiovascular parameters. These studies could help individuals with T1D to change their lifestyle to prevent or reduce the risk of cardiovascular disease in the long term with good diabetes management and regular physical activity.

## Data Availability

The original contributions presented in the study are included in the article/supplementary material. Further inquiries can be directed to the corresponding authors.

## References

[1] 9th edition | IDF diabetes atlas . Available online at: https://diabetesatlas.org/atlas/ninth-edition/ (Accessed onJanuary/19/2024).

[2] Lucier J Weinstock RS. Diabetes mellitus type 1. In: StatPearls. StatPearls Publishing, Treasure Island (FL (2023). Available at: http://www.ncbi.nlm.nih.gov/books/NBK507713/. [cited 2023 Apr 25].

[3] de Ferranti SD de Boer IH Fonseca V Fox CS Golden SH Lavie CJ. Type 1 diabetes mellitus and cardiovascular disease: A scientific statement from the American heart association and American diabetes association. Diabetes Care. (2014) 37:2843–63. https://www.ncbi.nlm.nih.gov/pmc/articles/PMC4170130/. 10.2337/dc14-1720 PMC4170130 25114297

[4] Orchard TJ Costacou T Kretowski A Nesto RW. Type 1 diabetes and coronary artery disease. Diabetes Care. (2006) 29:2528–38. doi: 10.2337/dc06-1161 17065698

[5] Broe R Rasmussen ML Frydkjaer-Olsen U Olsen BS Mortensen HB Hodgson L. Retinal vessel calibers predict long-term microvascular complications in type 1 diabetes: the Danish cohort of pediatric diabetes 1987 (DCPD1987). Diabetes. (2014) 63:3906–14. doi: 10.2337/db14-0227 24914239

[6] Nguyen TT Wang JJ Sharrett AR Islam FMA Klein R Klein BEK. Relationship of retinal vascular caliber with diabetes and retinopathy: the multi-ethnic study of atherosclerosis (MESA). Diabetes Care. (2008) 31:544–9. doi: 10.2337/dc07-1528 18070990

[7] Nguyen TT Wang JJ Islam FMA Mitchell P Tapp RJ Zimmet PZ. Retinal arteriolar narrowing predicts incidence of diabetes : the Australian diabetes, obesity and lifestyle (AusDiab) study. Diabetes. (2008) 57:536–9. doi: 10.2337/db07-1376 18086902

[8] Laurent S Cockcroft J Van Bortel L Boutouyrie P Giannattasio C Hayoz D. Expert consensus document on arterial stiffness: methodological issues and clinical applications. Eur Heart J. (2006) 27:2588–605. doi: 10.1093/eurheartj/ehl254 17000623

[9] Cheung CY Ikram MK Klein R Wong TY. The clinical implications of recent studies on the structure and function of the retinal microvasculature in diabetes. Diabetologia. (2015) 58:871–85. doi: 10.1007/s00125-015-3511-1 25669631

[10] Cristescu IE Zagrean L Balta F Branisteanu DC. Retinal microcirculation investigation in type I and II diabetic patients without retinopathy using an adaptive optics retinal camera. Acta Endocrinol (Buchar). (2019) 15:417–22. https://www.ncbi.nlm.nih.gov/pmc/articles/PMC7200121/. 10.4183/aeb.2019.417 PMC7200121 32377236

[11] Wu N Bredin SSD Guan Y Dickinson K Kim DD Chua Z. Cardiovascular health benefits of exercise training in persons living with type 1 diabetes: A systematic review and meta-analysis. J Clin Med. (2019) 8:253. https://www.ncbi.nlm.nih.gov/pmc/articles/PMC6406966/. 30781593 10.3390/jcm8020253 PMC6406966

[12] Way KL Lee AS Twigg SM Johnson NA. The effect of acute aerobic exercise on central arterial stiffness, wave reflections, and hemodynamics in adults with diabetes: A randomized cross-over design. J Sport Health Sci. (2021) 10:499–506. https://www.ncbi.nlm.nih.gov/pmc/articles/PMC8343005/. 32444343 10.1016/j.jshs.2020.02.009 PMC8343005

[13] Ansell SKD Jester M Tryggestad JB Short KR. A pilot study of the effects of a high-intensity aerobic exercise session on heart rate variability and arterial compliance in adolescents with or without type 1 diabetes. Pediatr Diabetes. (2020) 21:486–95. doi: 10.1111/pedi.12983 31951305

[14] Marshall ZA Mackintosh KA Lewis MJ Ellins EA McNarry MA. Association of physical activity metrics with indicators of cardiovascular function and control in children with and without type 1 diabetes. Pediatr Diabetes. (2021) 22:320–8. doi: 10.1111/pedi.13159 33215796

[15] Scott SN Cocks M Andrews RC Narendran P Purewal TS Cuthbertson DJ. High-intensity interval training improves aerobic capacity without a detrimental decline in blood glucose in people with type 1 diabetes. J Clin Endocrinol Metab. (2019) 104:604–12. doi: 10.1210/jc.2018-01309 30281094

[16] Tikellis G Anuradha S Klein R Wong TY. Association between physical activity and retinal microvascular signs: the Atherosclerosis Risk in Communities (ARIC) Study. Microcirculation. (2010) 17:381–93. doi: 10.1111/j.1549-8719.2010.00033.x PMC3005356 20618695

[17] Hanssen H Streese L Vilser W. Retinal vessel diameters and function in cardiovascular risk and disease. Prog Retinal Eye Res. (2022) 91:101095. https://www.sciencedirect.com/science/article/pii/S1350946222000556. 10.1016/j.preteyeres.2022.101095 35760749

[18] Kifley A Wang JJ Cugati S Wong TY Mitchell P. Retinal vascular caliber, diabetes, and retinopathy. Am J Ophthalmol. (2007) 143:1024–6. https://www.sciencedirect.com/science/article/pii/S0002939407001043. 10.1016/j.ajo.2007.01.034 17524767

[19] Tikellis G Wang JJ Tapp R Simpson R Mitchell P Zimmet PZ. The relationship of retinal vascular calibre to diabetes and retinopathy: the Australian Diabetes, Obesity and Lifestyle (AusDiab) study. Diabetologia. (2007) 50:2263–71. doi: 10.1007/s00125-007-0822-x 17891374

[20] Moser O Müller A Aberer F Aziz F Kojzar H Sourij C. Comparison of insulin glargine 300 U/mL and insulin degludec 100 U/mL around spontaneous exercise sessions in adults with type 1 diabetes: A randomized cross-over trial (ULTRAFLEXI-1 study). Diabetes Technol Ther. (2023) 25:161–8. doi: 10.1089/dia.2022.0422 36516429

[21] Saloň A Neshev R Teraž K Šimunič B Peskar M Marušič U. A pilot study: Exploring the influence of COVID-19 on cardiovascular physiology and retinal microcirculation. Microvascular Res. (2023) 150:104588. doi: 10.1016/j.mvr.2023.104588 37468091

[22] Mahdy A Stradner M Roessler A Brix B Lackner A Salon A. A pilot study: hypertension, endothelial dysfunction and retinal microvasculature in rheumatic autoimmune diseases. J Clin Med. (2021) 10:4067. doi: 10.3390/jcm10184067 34575178 PMC8467719

[23] Stoner L Young JM Fryer S. Assessments of arterial stiffness and endothelial function using pulse wave analysis. Int J Vasc Med. (2012) 2012:903107. doi: 10.1155/2012/903107 22666595 PMC3361177

[24] Saloň A Steuber B Neshev R Schmid-Zalaudek K De Boever P Bergmann E. Vascular responses following light therapy: A pilot study with healthy volunteers. J Clin Med. (2023) 12:2229. doi: 10.3390/jcm12062229 36983231 PMC10054429

[25] Khan A Boever PD Gerrits N Akhtar N Saqqur M Ponirakis G. Retinal vessel multifractals predict pial collateral status in patients with acute ischemic stroke. PloS One. (2022) 17:e0267837. doi: 10.1371/journal.pone.0267837 35511879 PMC9070887

[26] Knudtson MD Lee KE Hubbard LD Wong TY Klein R Klein BEK. Revised formulas for summarizing retinal vessel diameters. Curr Eye Res. (2003) 27:143–9. doi: 10.1076/ceyr.27.3.143.16049 14562179

[27] Marshall ZA Mackintosh KA Gregory JW McNarry MA. Using compositional analysis to explore the relationship between physical activity and cardiovascular health in children and adolescents with and without type 1 diabetes. Pediatr Diabetes. (2022) 23:115–25. doi: 10.1111/pedi.13288 34780103

[28] Brazeau AS Gingras V Leroux C Suppère C Mircescu H Desjardins K. A pilot program for physical exercise promotion in adults with type 1 diabetes: the PEP-1 program. Appl Physiol Nutr Metab. (2014) 39:465–71. doi: 10.1139/apnm-2013-0287 24669988

[29] Gusso S Pinto T Baldi JC Derraik JGB Cutfield WS Hornung T. Exercise training improves but does not normalize left ventricular systolic and diastolic function in adolescents with type 1 diabetes. Diabetes Care. (2017) 40:1264–72. doi: 10.2337/dc16-2347 28720592

[30] Hanssen H Nickel T Drexel V Hertel G Emslander I Sisic Z. Exercise-induced alterations of retinal vessel diameters and cardiovascular risk reduction in obesity. Atherosclerosis. (2011) 216:433–9. https://www.sciencedirect.com/science/article/pii/S0021915011001651. 10.1016/j.atherosclerosis.2011.02.009 21392768

[31] Siegrist M Hanssen H Lammel C Haller B Koch AM Stemp P. Effects of a cluster-randomized school-based prevention program on physical activity and microvascular function (JuvenTUM 3). Atherosclerosis. (2018) 278:73–81. doi: 10.1016/j.atherosclerosis.2018.09.003 30261471

[32] Ludyga S Köchli S Pühse U Gerber M Hanssen H. Effects of a school-based physical activity program on retinal microcirculation and cognitive function in adolescents. J Sci Med Sport. (2019) 22:672–6. doi: 10.1016/j.jsams.2018.11.029 30553766

[33] Streese L Khan AW Deiseroth A Hussain S Suades R Tiaden A. High-intensity interval training modulates retinal microvascular phenotype and DNA methylation of p66Shc gene: a randomized controlled trial (EXAMIN AGE). Eur Heart J. (2020) 41:1514–9. doi: 10.1093/eurheartj/ehz196 31323685

[34] Anuradha S Healy GN Dunstan DW Klein R Klein BE Cotch MF. Physical activity, television viewing time, and retinal microvascular caliber: the multi-ethnic study of atherosclerosis. Am J Epidemiol. (2011) 173:518–25. doi: 10.1093/aje/kwq412 PMC3105441 21300854

[35] Salem MA AboElAsrar MA Elbarbary NS ElHilaly RA Refaat YM. Is exercise a therapeutic tool for improvement of cardiovascular risk factors in adolescents with type 1 diabetes mellitus? A randomised controlled trial. Diabetol Metab Syndr. (2010) 2:47. https://www.ncbi.nlm.nih.gov/pmc/articles/PMC3238209/. 20618996 10.1186/1758-5996-2-47 PMC3238209

[36] Giacco F Brownlee M. Oxidative stress and diabetic complications. Circ Res. (2010) 107:1058–70. https://www.ncbi.nlm.nih.gov/pmc/articles/PMC2996922/. 10.1161/CIRCRESAHA.110.223545 PMC2996922 21030723

[37] Beltramo E Porta M. Pericyte loss in diabetic retinopathy: mechanisms and consequences. Curr Med Chem. (2013) 20:3218–25. doi: 10.2174/09298673113209990022 23745544

[38] Blum M Brändel C Müller UA. Myogenic response reduction by high blood glucose levels in human retinal arterioles. Eur J Ophthalmol. (2005) 15:56–61. doi: 10.1177/112067210501500109 15751240

[39] Cipolla MJ Porter JM Osol G. High glucose concentrations dilate cerebral arteries and diminish myogenic tone through an endothelial mechanism. Stroke. (1997) 28:405–11. doi: 10.1161/01.STR.28.2.405 9040698

[40] Liu M Lovern C Lycett K He M Wake M Wong TY. The association between markers of inflammation and retinal microvascular parameters: A systematic review and meta-analysis. Atherosclerosis. (2021) 336:12–22. https://www.sciencedirect.com/science/article/pii/S0021915021013654. 34607278 10.1016/j.atherosclerosis.2021.09.025

[41] Streese L Guerini C Bühlmayer L Lona G Hauser C Bade S. Physical activity and exercise improve retinal microvascular health as a biomarker of cardiovascular risk: A systematic review. Atherosclerosis. (2020) 315:33–42. doi: 10.1016/j.atherosclerosis.2020.09.017 33212315

